# PAK4 regulates G6PD activity by p53 degradation involving colon cancer cell growth

**DOI:** 10.1038/cddis.2017.85

**Published:** 2017-05-25

**Authors:** Xiumei Zhang, Xia Zhang, Yang Li, Yangguang Shao, Jianying Xiao, Ge Zhu, Feng Li

**Affiliations:** 1 Department of Cell Biology, Key Laboratory of Cell Biology, Ministry of Public Health, and Key Laboratory of Medical Cell Biology, Ministry of Education, China Medical University, Shenyang, Liaoning, China; 2 Department of Biochemistry and Molecular Biology, Jinzhou Medical University, Jinzhou, Liaoning, China

## Abstract

The p21-activated kinase 4 (PAK4) is overexpressed in different cancers and promotes proliferation of cancer cells. Reprogramming of glucose metabolism is found in most cancer cells which in turn supports rapid proliferation. However, the relationship between PAK4 and glucose metabolism in cancer cells has not been explored. In this study, we reported that PAK4 promoted glucose intake, NADPH production and lipid biosynthesis, leading to an increased proliferation of colon cancer cells. Mechanistically, PAK4 interacted with glucose-6-phosphate dehydrogenase (G6PD), a rate-limiting enzyme of the pentose phosphate pathway and increased G6PD activity via enhancing Mdm2-mediated p53 ubiquitination degradation. In addition, we demonstrated a close positive correlation between PAK4 and G6PD expression in colon cancer specimens. Furthermore, expression of PAK4 or G6PD was positively correlated with an aggressive phenotype of clinical colon cancer. These findings revealed a novel glucose metabolism-related mechanism of PAK4 in promoting colon cancer cell growth, suggesting that PAK4 and/or G6PD blockage might be a potential therapeutic strategy for colon cancer.

Several evidences confirmed that most of the cancer cells consume more glucose and create a metabolic reprogramming that is essential for quick proliferation and survival, through substantial alterations in several energy metabolism pathways, including glucose transport, glycolysis and pentose phosphate pathways (PPP).^1, 2, 3^ Alterations in glucose metabolism of cancer cells is directly regulated by several oncogenic pathways, including the PI3K/Akt, Myc, or hypoxia-inducible factor (HIF) pathways which serve to increase the glycolysis and consecutively promotes cell proliferation.^4, 5, 6^

The p21-activated kinases (PAKs) are a family of serine/threonine protein kinases, which are classified into two groups as Group I (PAK1–3) and Group II (PAK4–6).^7, 8, 9^ All PAKs are often overexpressed in a variety of tumors and play an important role in the cytoskeletal reorganization, cell survival, gene transcription and cell transformation.^10, 11^ PAK4, a representative of Group II, is involved in the tumorigenesis and progression^12, 13^ through promoting growth and proliferation^14, 15^ as well as migration and metastasis.^16, 17^ However, whether PAK4 regulates glucose metabolism in tumor cells remains to be elucidated.

Due to the pivotal role of PAK4 as key regulator in cancer cell signaling networks, we sought to specifically probe the role of PAK4 in regulating the colon cancer cell metabolism and proliferation.

## Results

### PAK4 promotes the production of cellular lipids and other metabolites

**Figure 1:**
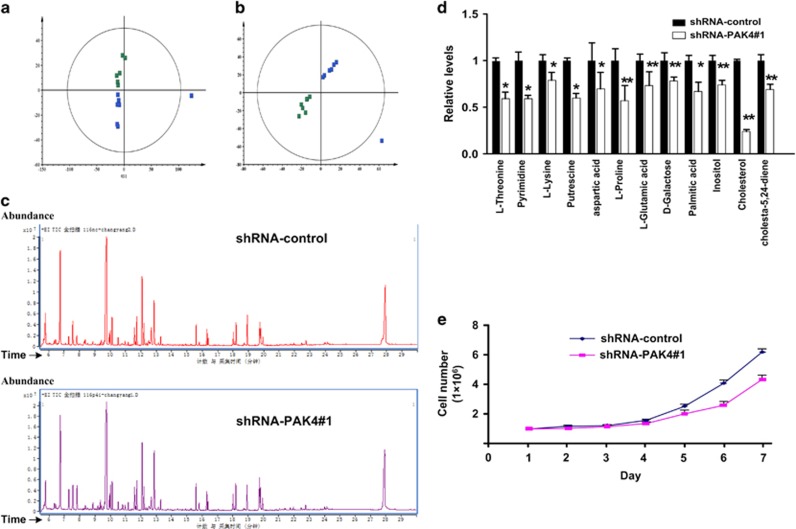
Metabolic Profiles of PAK4 silencing in HCT-116 p53+/+ cells. (**a**) The PCA scores plot based on GC–MS of cells showed that different samples were scattered into two different regions. Green box (□); shRNA-control: blue diamond (◊), shRNA-PAK4. (**b**) PLS-DA scores plot based on GC–MS of cells obtained from different groups. Green box (□); shRNA-control: blue diamond (◊), shRNA-PAK4. (**c**) Representative GC/MS ion chromatograms of the samples from shRNA-control and shRNA-PAK4 groups (**d**) Differential metabolites between shRNA-PAK4 and shRNA-control in HCT-116 p53+/+ cells. (**e**) Growth curves of PAK4 silencing and control HCT-116 p53 +/+ cells (*n*=3) were cultured in medium containing glucose. Average of three independent experiments was shown

It has been shown that PAK1 is a regulator of glucose metabolism.^18, 19, 20^ We hypothesized that PAK4, a representative of Group II, could also serve as an important regulator of glucose metabolism which in turn regulates tumor cell growth and proliferation. Gas chromatography–mass spectrometry (GC–MS) was performed to examine the influence of PAK4 silencing on metabolites of HCT-116 p53+/+ cells. The efficacy of PAK4-shRNA was demonstrated by depleting PAK4 (Supplementary Figure 1b). Then a principal component analysis (PCA) model, an unsupervised projection method, was constructed and then visualized the dataset to display the similarities and differences. The PCA scores were plotted which showed scattering of different samples in two different regions (Figure 1a). Further research by partial least squares-discriminant analysis (PLS-DA), a supervised projection method, showed that sample points were completely separated, which indicated that the metabolites are different between PAK4 silencing cells and PAK4 control counterparts (Figure 1b). Representative GC/MS total ion chromatograms (TICs) of paired samples of shRNA-control and shRNA-PAK4 groups were displayed (Figure 1c). Differential metabolites were further identified and validated by searching the online databases between the two groups (Table 1). Silencing of PAK4 resulted in a significant decrease in palmitic acid and cholesterol production (Figure 1d). Furthermore, PAK4 knockdown also declined other metabolites, such as 5–24 diene cholesteric, pyrimidine, putrescine, aspartic acid, threonine, proline, glutamic acid, lysine, inositol, galactose and so on (Figure 1d). These results suggested that PAK4 may be associated with lipid biosynthesis. Because the raw materials of lipid biosynthesis primarily come from glucose, so we hypothesized that PAK4 overexpression in colon cancer cells may use lipid biosynthesis to support the increased proliferation by directing glucose towards the biosynthetic processes. Indeed, PAK4 silencing cells grew significantly slower than the control cells (Figure 1e).

### PAK4 promotes consumption of glucose and NADPH production

**Figure 2:**
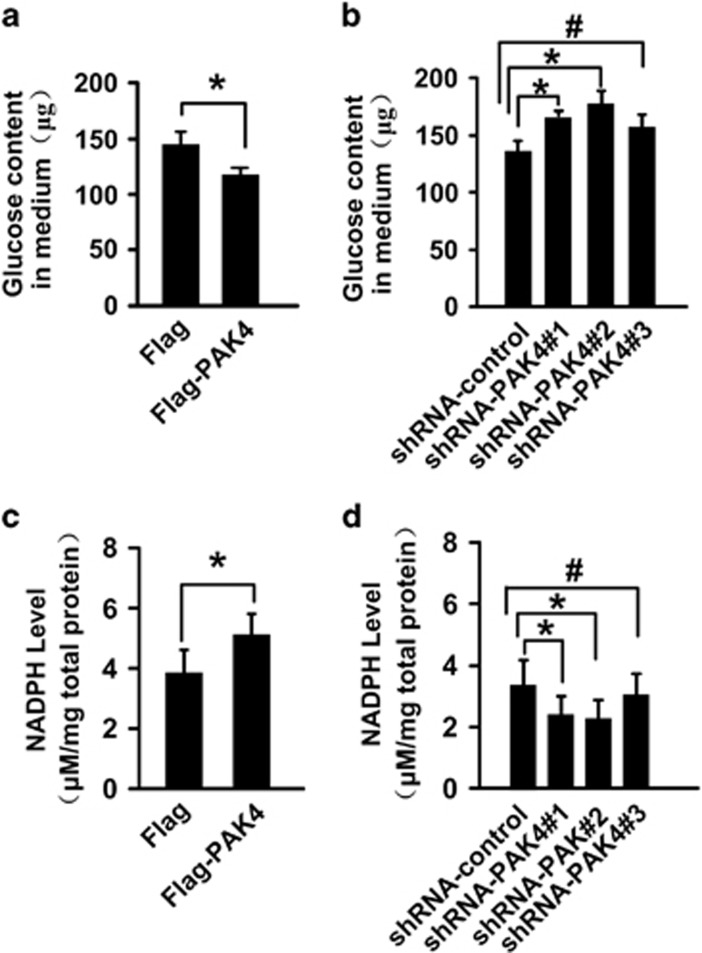
PAK4 promotes consumption of glucose and NADPH production. (**a** and **b**) The content of glucose in the culture medium was measured in HCT-116 p53+/+ cells with PAK4 overexpression or PAK4 knockdown, respectively. (**c** and **d**) The NADPH production was measured in HCT-116 p53+/+ cells with PAK4 overexpression or PAK4 knockdown, respectively. The results were expressed as mean±S.D. of three independent experiments. *P<0. 05, ^#^*P*>0. 05

NADPH is required for the biosynthesis of lipids (such as fatty acids and cholesterol) and primarily from glucose catabolism in mammal cells. Then we investigated whether PAK4 regulates the glucose consumption and NADPH production. HCT-116 p53+/+ cells were transfected with PAK4 plasmid and then were stably transfected with different lentiviral vectors shRNA-PAK4 and shRNA-control. The efficacy of PAK4 overexpression and knockdown were demonstrated by increased and reduced PAK4 expression. Moreover, depletion of PAK4 does not affect PAK1 protein expression levels (Supplementary Figures 1a–c). The data showed that the glucose content in the medium was decreased with PAK4 overexpression (Figure 2a), and increased with PAK4 knockdown in HCT-116 p53+/+ cells (Figure 2b). The results indicated that PAK4 promoted consumption of glucose. Moreover, PAK4 overexpression lead to an increase in the NADPH level in HCT-116 p53+/+ cells (Figure 2c), while PAK4 knockdown in HCT-116 p53+/+ cells with small hairpin RNA (shRNA) decreased the production of NADPH (Figure 2d).

### PAK4 regulation of G6PD activity

**Figure 3:**
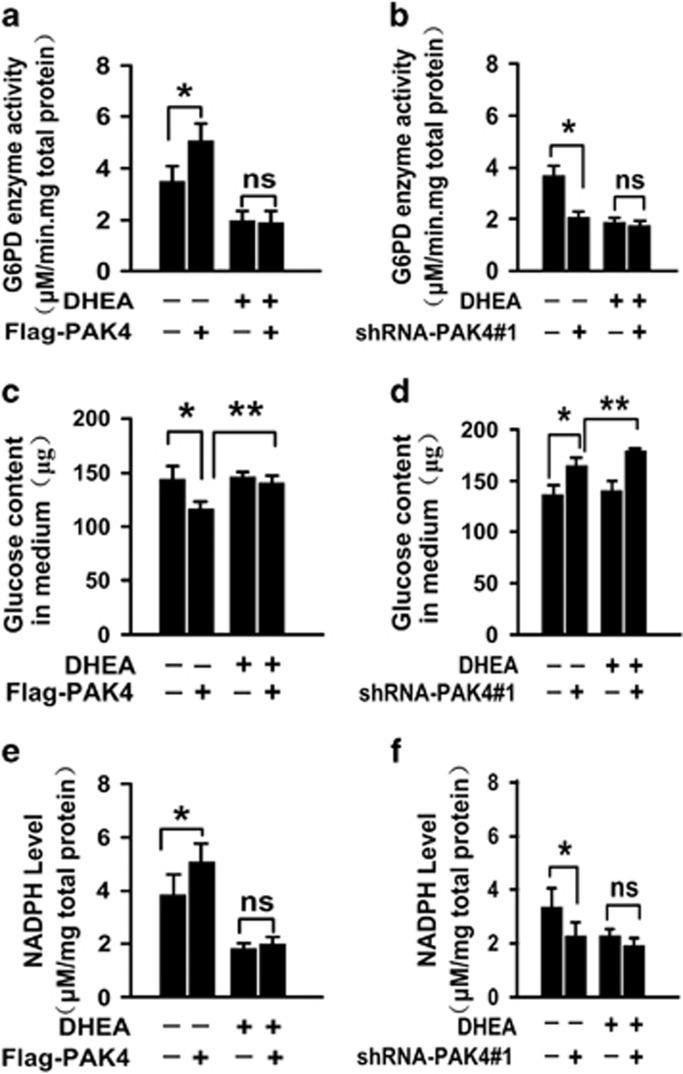
PAK4 increases G6PD activity. (**a** and **b**) HCT-116 p53+/+ cells overexpression or knockdown of PAK4 were treated with 1 mM DHEA or vehicle for 24 h. Then the G6PD enzyme activity was measured. (**c** and **d**) HCT-116 p53+/+ cells with overexpression or knockdown of PAK4 were treated with 1 mM DHEA or vehicle (−) for 24 h, then the glucose consumption was measured. (**e** and **f**) HCT-116 p53+/+ cells overexpression or knockdown of PAK4 were treated with 1 mM DHEA or vehicle for 24 h, then the NADPH production was measured. All the results were presented as means±S.D. of three independent experiments. *P<0. 05, **P<0. 01, NS indicates no significance

In order to investigate the mechanism of PAK4 in glucose consumption and NADPH production, we assayed the activity of G6PD, a rate-limiting enzyme of PPP pathway. PPP pathway is considered as the main pathway in the production of NADPH. Overexpression of PAK4 was correlated with a strong elevation in G6PD activity in HCT-116 p53+/+ cells. Conversely, PAK4 knockdown caused a noticeable decrease in the G6PD activity. Treatment with DHEA (dehydroepiandrosterone), an inhibitor of G6PD enzyme activity, minimized the difference between G6PD activity in HCT-116 p53+/+ cells with PAK4 overexpression or knockdown (Figures 3a and b). Moreover, inhibition of G6PD activity using DHEA in HCT-116 p53+/+ cells reversed the glucose consumption (Figures 3c and d) and NADPH production caused by PAK4 (Figures 3e and f). Taken together, these results suggested that PAK4 promoted glucose consumption and NADPH production in colon cancer cells via increasing the enzyme activity of G6PD.

### G6PD is identified as a novel binding partner of PAK4

**Figure 4:**
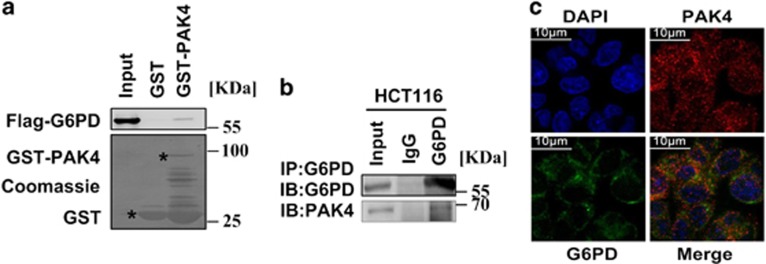
Identification of G6PD as an interacting protein of PAK4. (**a**) G6PD directly binds to the GST-tagged PAK4 *in vitro*. For GST pull-down assay, GST or GST-PAK4 fusion proteins were incubated with Flag-G6PD which were translated *in vitro*. Bound proteins were detected by western blotting. Black stars indicate the GST or GST-fusion protein. (**b**) Endogenous G6PD interacts with PAK4. HCT-116 cells lysates were immunoprecipitated with the indicated antibody or IgG. Precipitates were analyzed by western blotting using the indicated antibodies. (**c**) HCT-116 cells were fixed and then incubated with anti-PAK4 and anti-G6PD antibodies. Alexa Fluor 488 (green) or Alexa Fluor 546 (red) was used to detect G6PD and PAK4 respectively; nucleus was stained with DAPI (4, 6-diamidino-2-phenylindole). Yellow indicates co-localization. Original magnification was × 600

According to the previous studies, it was confirmed that PAK4 regulates the G6PD activity. Firstly, to verify the potential of PAK4-G6PD interaction, we performed *in vitro* glutathione S-transferase (GST)-binding assay. The results showed that an *in vitro* translated G6PD interaction with GST-PAK4 (Figure 4a). Importantly, immunoprecipitation of endogenous G6PD of HCT-116 p53+/+ cells also pulled down PAK4 protein using G6PD-specific antibody (Figure 4b). To further characterize the interaction between PAK4 and G6PD, we examined the co-localization of endogenous G6PD with endogenous PAK4 in HCT-116 p53+/+ cells. It was observed that G6PD co-localized with PAK4 in the cytoplasm (Figure 4c). Together, these data indicated that G6PD was a PAK4-binding protein in colon cancer cells. As PAK4 kinase acts on its targets mainly through phosphorylation, we next investigated whether PAK4 phosphorylated G6PD. The results demonstrated no phosphorylation of GST-G6PD by PAK4 (Supplementary Figures 2a, lane 4).

### PAK4 promotes ubiquitination-mediated p53 degradation by enhancing the binding of murine double minute 2 and p53

**Figure 5:**
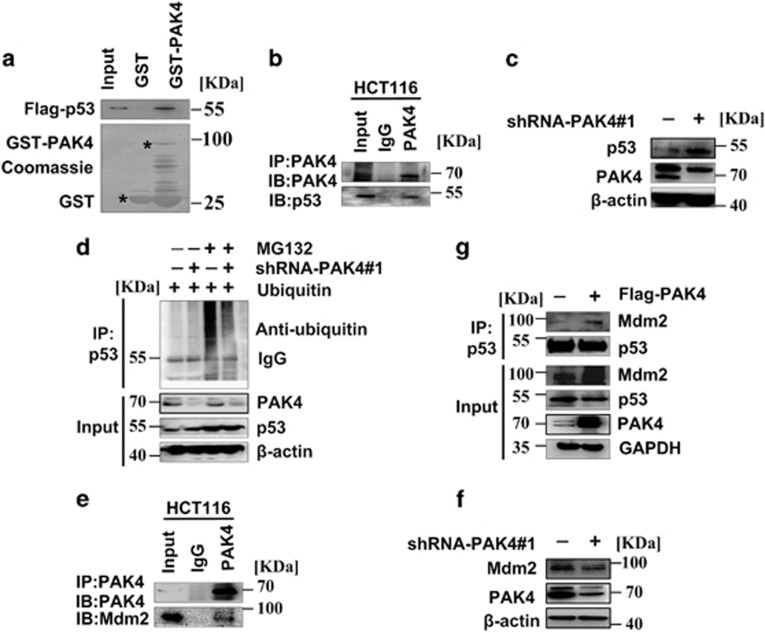
PAK4 promotes ubiquitination-mediated p53 degradation via enhancing the binding of p53 and Mdm2. (**a**) p53 directly binds to the GST-tagged PAK4 *in vitro*. GST pull-down assay showed that GST or GST-PAK4 fusion proteins were incubated with Flag-p53 translated plasmid *in vitro*. Bound proteins were detected with western blotting. Black stars indicate GST and GST-fusion protein. (**b**) Endogenous p53 interacts with PAK4. HCT-116 p53+/+ cells were treated with 5 nM DOX for 12 h. Total lysates were immunoprecipitated with PAK4 antibody or IgG. Precipitates were analyzed by western blotting using the indicated antibodies. (**c** and **f**) HCT-116 p53+/+ cells were stably transfected with lentiviral PAK4-shRNA#1 and the control shRNA (Ctrl shRNA). Cell lysates from the cells were used for immunoblotting analysis using the indicated antibodies. (**d**) PAK4 promotes p53 ubiquitination.HCT-116 p53+/+cells were co-transfected with the indicated constructs for 24 h and then were treated by 5 mM MG132 or had no treatment. Total lysates were subjected to immunoprecipitation and western blotting with the indicated antibodies. (**e**) Endogenous PAK4 interacts with Mdm2. HCT-116 p53+/+ cells lysates were immunoprecipitated with PAK4 antibody or IgG. Precipitates were analyzed by western blotting using the indicated antibodies. (**g**) PAK4 enhances the binding of p53 and Mdm2. COS7 cells were co-transfected with Flag-PAK4 or control vector for 24 h, 30 *μ*g of total cell lysates were set aside as input; equal amounts of protein lysates were subjected to immunoprecipitation (IP) and immunoblot (IB) analysis with the indicated antibodies

Then the study explored the mechanism by which PAK4 regulates the G6PD activity. It has been reported that p53 directly inactivated G6PD^21^ and p53 is the downstream protein of PAK4,^14^ then we speculated whether PAK4 affects the activity of G6PD through p53. To verify the potential of PAK4-p53 interaction, we performed *in vitro* GST-binding assay. The results showed that *in vitro* translated p53 binds with GST-PAK4 (Figure 5a). Moreover, the results of immunoprecipitation assay indicated that PAK4 could interact with p53 (Figure 5b). We then examined whether PAK4 might regulate the protein level of p53. PAK4 silencing increased the protein level of p53 (Figure 5c) and previous studies have reported that protein levels of p53 were modulated by proteasome-dependent degradation.^22^ To validate whether PAK4 regulates p53 protein level through this process, the 26 S proteasome inhibitor MG132 was used. Ubiquitination assay showed that the ubiquitination of p53 was impaired with PAK4 knockdown (Figure 5d). Previous studies have demonstrated that murine double minute 2 (Mdm2) interacts with p53 and promoted p53 degradation via its E3 ligase activity.^23^ To test whether Mdm2 was involved in the PAK4-dependent promotion of p53 ubiquitination, we performed immunoprecipitation and western blotting analysis. First, we found that Mdm2 interacted with PAK4 (Figure 5e). Then, we observed lentivirus-mediated knockdown of PAK4 decreased the Mdm2 expression in HCT-116 p53+/+ cells (Figure 5f). Importantly, PAK4 promoted the binding of Mdm2 and p53 (Figure 5g). Taken together, these data suggested that PAK4 promoted ubiquitination-mediated p53 degradation by enhancing the binding of Mdm2 and p53.

### PAK4 hardly affects the consumption of glucose and NADPH production in HCT-116 p53−/− cells

**Figure 6:**
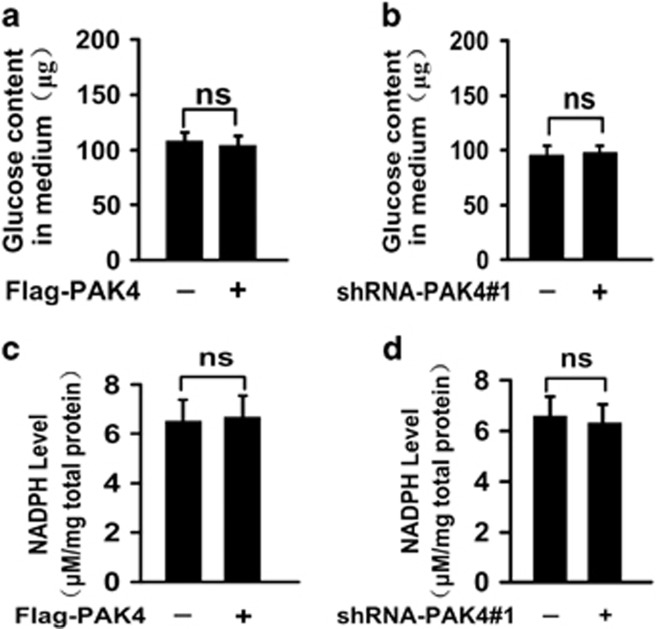
PAK4 does not promote consumption of glucose and NADPH production in HCT-116 p53−/− cells. (**a** and **b**) The glucose content in the culture medium was measured in HCT-116 p53−/− cells with PAK4 overexpression and PAK4 knockdown, respectively. (**c** and **d**) NADPH production was measured in HCT-116 p53−/− cells with PAK4 overexpression and PAK4 knockdown using shRNA, respectively. All the results were expressed as mean±S.D. of three independent experiments, NS indicates no significance

In order to affirm the above mechanism, we also explored the glucose consumption and NADPH production in HCT-116 p53−/− cells. The results showed that the glucose consumption barely changed with PAK4 overexpression and knockdown in HCT-116 p53−/− cells (Figures 6a and b). PAK4 overexpression and knockdown also barely affected the NADPH levels in HCT-116 p53−/− cells (Figures 6c and d). The results indicated that PAK4 regulation of consumption of glucose and NADPH production was dependent on p53 status.

### PAK4 correlates with G6PD expression in colon cancer tissue samples

**Figure 7:**
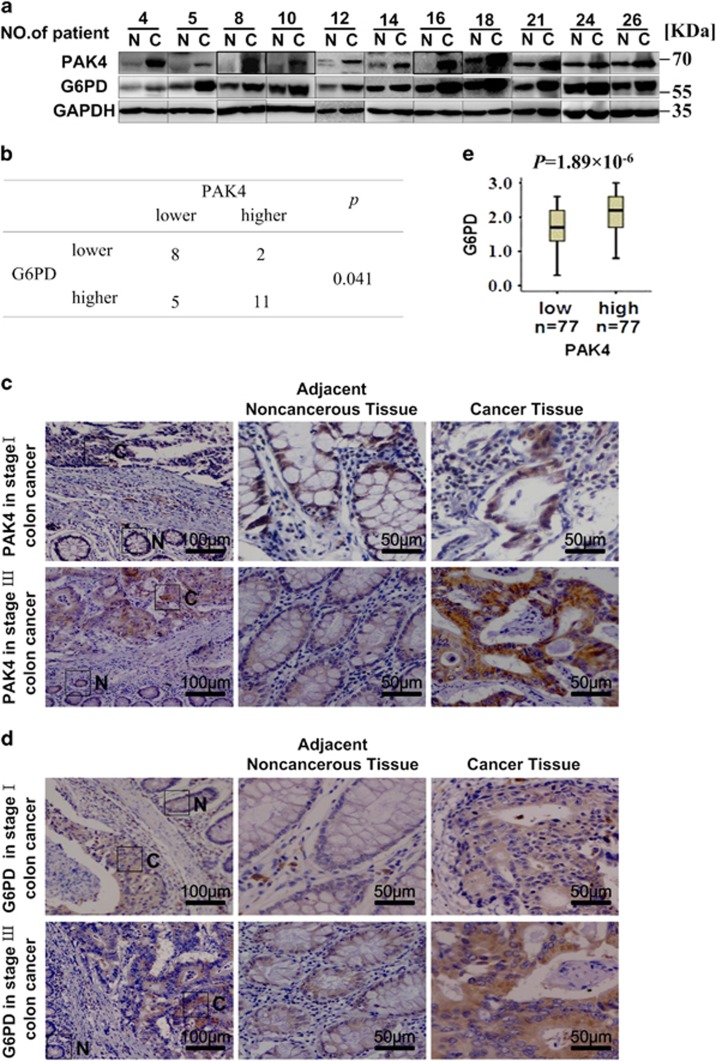
Correlation of PAK4 and G6PD in colon cancer. (**a**) The protein levels of PAK4 and G6PD were examined in colon cancer cells. N, matched adjacent noncancerous mucosa. C, colon cancer tissue. GAPDH served as protein loading control. The representative 11 pairs are shown, and all the results are shown in Supplementary Figure 3. (**b**) Summary of tissue expressions in a was shown, with tissues categorized by lower and higher expression. The expression of PAK4 and G6PD was analyzed with glyceraldehyde 3-phosphate dehydrogenase as reference. In each N and C pair, the lower/higher expression in C, compared with N, was categorized as lower/higher expression. *P*-value was generated using the *χ*^2^-test. (**c** and **d**) Representative images of immunohistochemical staining from two different stage cases. The boxed areas on the left images are magnified in the middle and right images. N, adjacent noncancerous tissue (shown in the middle column); C, cancer tissue (shown in the right column). Original magnification of left images was × 100. Intensity values are expressed as HSCOREs, as specified in materials and methods. Data were analyzed by Mann–Whitney *U*-test, as shown in Tables 1 and 2. (**e**) Box plot of G6PD expression. The subjects were divided into two groups based on PAK4 expression scores in the tumors, representing low and high expression levels. The G6PD expression scores are shown as box plots with horizontal lines representing the median; the bottom and top of the boxes representing the 25th and 75th percentiles, respectively; and the vertical bars representing the range of data

In order to further explore the role of PAK4 in colon cancer cells as well as to substantiate the functional link between PAK4 and G6PD, protein levels of PAK4 and G6PD were examined by western blotting in 26 pairs of colon cancer tissue specimens and matched the adjacent noncancerous tissue specimens. The results showed that PAK4 expression was positively correlated with G6PD expression (*P*=0.041) in colon cancer tissues (Supplementary Figure 3). Representative pictures were shown in Figures 7a and b. PAK4 and G6PD were highly expressed in 50% (13/26) and 61.5% (16/26) of colon cancer samples, respectively. The levels of G6PD and PAK4 were increased in colon cancer tissue samples compared with adjacent noncancerous tissue samples. The results were consistent with our findings that PAK4 upregulated G6PD activity in colon cancer cell lines.

We also performed immunohistochemical staining of PAK4 and G6PD in 154 cases with colon cancer to confirm this observation. Representative images were shown in Figures 7c and d. The immunoreactivity of G6PD and PAK4 was too low to be detected in the adjacent noncancerous tissues (Figures 7c and d, middle). Unlike noncancerous tissues, high expression levels of G6PD and PAK4 were observed in colon cancer tissues (Figures 7c and d, right). To better understand the correlation between the expression of PAK4 or G6PD and progression of colon cancer, these samples were divided into two groups based on PAK4 or G6PD levels (histological score). The data showed that high expression of PAK4 or G6PD was significantly associated with poor pTNM stage (*P*=0.02 or *P*=0.037, respectively) and histological grade (*P*=0.003 or *P*=0.042, respectively), (Tables 2 or 3), but not with tumor size (*P*=0.932 or *P*=0.649, respectively) and depth of invasion (pT), (*P*=0.656 or *P*=0.114, respectively). In order to better understand the relationship between the expression scores of PAK4 and G6PD scores, we divided the samples into two groups on the basis of PAK4 levels (cut off at the median score) and studied the differences of G6PD expression. The data showed that the expression scores of G6PD in tumors were consistent with PAK4 scores (*P*=1.89 × 10^−6^; Figure 7e). These results were in consistent with our *in vitro* observations, which suggested that PAK4 was overexpressed in colon cancer tissues, leading to the increased G6PD activity.

## Discussion

Reprogramming of glucose metabolism is one of the key characteristic feature of cancer cells to coordinate glucose utilization with cell physiology. One of the mechanism used by cancer cells to support the rapid proliferation is the redirection of glucose towards the PPP, which may be beneficial to the biosynthesis.^21, 24, 25^ The first rate-limiting enzyme of PPP is G6PD, which activity and location are regulated by p53, BAG3, HGF and AMP Kinase.^21, 25, 26^ The current study demonstrated that PAK4 directly interacted with G6PD (Figure 4), increased G6PD activity in HCT-116 p53+/+ (Figure 3), and promoted glucose consumption, NADPH production (Figure 2) and lipid biosynthesis (Figure 1), leading to a significant increase in colon cancer cell proliferation (Figure 1). PAK4 works via both kinase-dependent and kinase-independent mechanisms.^27^ We recently demonstrated an oncogenic role of PAK4 in the repression of TGF-*β*-mediated growth inhibition in gastric cancer cells. PAK4 interacts with Smad2/3 by a kinase-independent mechanism, and then blocks the TGF-*β*-induced phosphorylation of Smad2 or Smad3 as well as their activation. PAK4 also phosphorylates Smad2 at Ser465 by a kinase-dependent mechanism, leading to the degradation of Smad2 through the ubiquitin–proteasome pathway under hepatocyte growth factor (HGF) stimulation.^12^ In this study, we found that PAK4 could not phosphorylate G6PD (Supplementary Figure 2, lane 4), which led us to study the mechanism of PAK4 regulation of G6PD activity.

It is reported that PAK4 acts as upstream regulators of p53, and significantly reduces camptothecin-induced phospho-S474-PAK4 and p53 levels.^14^ However, downregulation of PAK4 causes S phase arrest and is associated with the upregulation of p53 in Hep-2 laryngeal carcinoma cells.^28^ Thus, further definition and mapping of the link between PAK4 and p53 signaling may have important implications in the treatment of PAK4-related cancers. In the present study, we demonstrate that PAK4 interacts with p53 and downregulates p53 protein level (Figures 5a–c). It is reported that p53 interacts with G6PD and could inhibit G6PD activity indirectly.^21^ Therefore, we speculate that PAK4 may increase G6PD activity through downregulation of p53. p53 protein stability was mainly regulated by ubiquitin-dependent proteasomal degradation pathway.^22^ Mdm2, the principal cellular antagonist of p53, has long been believed to promote p53 degradation via its E3 ligase activity.^23^ Notably, our data showed that PAK4 promotes the binding of Mdm2 and p53 (Figure 5g). Moreover, PAK4 markedly enhances the ubiquitination of p53 (Figure 5d). These results indicate that PAK4 increases G6PD activity via enhancing Mdm2-mediated p53 ubiquitination degradation. In HCT-116 p53−/− cells, PAK4 scarcely affect the glucose consumption and NADPH production (Figure 6). The results affirmed the above mechanism consecutively.

Overexpression of PAK4 has been detected in human colon cancer cell lines^29^ as well as in primary mouse colon tumors.^30^ PAK4 gene was also observed to be amplified in CRC patient samples.^31^ Moreover, PAK4 is essential for HCT-116 colon cancer cell proliferation in anchorage-independent culture.^29^ It has been recently reported that PAK4 expression is associated with colorectal cancer infiltration and metastasis.^32^ In consistent with the previous studies, our results revealed that PAK4 promoted colon cancer cell growth (Figure 1e). We also found that PAK4 level was significantly increased in 113 out of 154 patients with colon cancer. High expression of PAK4 was associated with poor pTNM stage and histological grade (Table 2).

According to the recently available data, G6PD is an oncogene that upregulated in various tumors, including bladder cancer,^33^ breast cancer,^34, 35, 36^ esophageal squamous cell carcinoma.^37, 38^ Overexpression of G6PD was closely associated with the progression of gastric cancer, and might be regarded as an independent predictor for the poor prognosis of gastric cancer.^39^ However, the expression and significance of G6PD in human colon cancer progression still remains unclear. In the present study, we identified elevated expression levels of G6PD in colon cancer tissues compared with noncancerous tissues in 100 out of 154 colon cancer patients. We also demonstrate that increased G6PD expression was associated with aggressive colon cancer behavior (Table 3). Importantly, for the first time, we established a correlation between PAK4 and G6PD in colon cancer with clinicopathological analysis.

In summary, this is the first evidence that PAK4 increased G6PD activity via enhancing Mdm2-mediated p53 ubiquitination degradation, and thus promoted the glucose intake, NADPH, palmitic acid, cholesterol and other metabolites production of colon cancer cells, leading to the increased colon cancer cell growth. Notably, we showed that there was a strong positive correlation between PAK4 and G6PD expression in colon cancer specimens and that the expression of PAK4 or G6PD was positively correlated with an aggressive phenotype of clinical colon cancer. We demonstrate a novel glucose metabolism-related mechanism of PAK4 in promoting the colon cancer cell growth. Therefore, inhibition of PAK4 and/or G6PD activity might be a potential therapeutic strategy for colon cancer.

## Materials and methods

### Cell culture

Human colon cancer cell lines (HCT116p53^+/+^ and HCT116p53^−/−^), human kidney 293 cells (HEK-293), COS7 cells were used in our experiments. HCT-116 p53−/−, HEK-293 cells and COS7 cells were maintained in Dulbecco’s modified Eagle’s medium (Gibco, Los Angeles, CA, USA). HCT116p53+/+ cells were propagated in RPMI 1640 medium (Gibco). All media were supplemented with 10% fetal bovine serum, and all cell lines were cultured at 37 °C in a humidified incubator at 5% CO_2_.

### Plasmid construction

Gv219-G6PD vector was purchased from GeneChem Company (Shanghai, China) and amplification was performed by PCR and inserted into pcDNA3.1(Hygro)-Flag and PGEX-4T-2. Flag-PAK4 and Flag-p53 plasmids were constructed and used as described previously.^17^

### Lentiviral vector production and generation of stable cell lines

PAK4-RNAi-lentivirus were purchased from Shanghai GeneChem Company (Shanghai, China). The shRNA-PAK4#1 sequence was 5′-CTTCATCAAGATTGGCGAG-3′, The shRNA-PAK4#2 sequence was 5′- CTAAGAGGTGAACATGTAT-3′, The shRNA-PAK4#3 sequence was 5′-GGATGAACGAGGAGCAGAT-3′ and shRNA-control sequence was 5′-UUCUCCGAACGUGUCACGUTT-3′.^16^

Commercial lentivirus was used to infect HCT-116 cells in a 12-well plate and stable clonal cell lines were selected with 2 *μ*g/ml puromycin. Infected HCT-116 cells were identified by western blotting.

### Immunoprecipitation, western blotting analysis and GST pull-down assays

Total protein was extracted from HCT-116 cells and protein concentration was measured using a BCA Protein Assay Kit (KeyGen). Total protein lysate (2 mg) was used for each immunoprecipitation (IP) using specific antibody or the corresponding IgG control. Protein A agarose beads (GE Healthcare, Uppsala, Sweden) were added to the cells and then were incubated overnight at 4 °C. Washed precipitated proteins were analyzed by western blotting. The IP, western blotting and GST pull-down assays were used in this study as described previously in detail.^40^

### Immunofluorescence

Immunofluorescence analysis has been described previously.^41^ Images were acquired using an Olympus fluorescence microscope (Olympus, Tokyo, Japan).

### Kinase assay

Kinase assay has been described previously.^13^ Recombinant PAK4 kinase was used for kinase assay. The GST-fusion proteins were stained using Ponceau or Coomassie brilliant blue.

### Glucose consumption

The cultured cells were transfected with PAK4-short hairpin RNA and Flag-PAK4 plasmid and cultured for 6 h.The culture medium was then changed and cells were incubated for an additional 24 h. Glucose levels in the culture medium were measured using the Glucose (GO) assay kit (Sigma, St. Louis, MO, USA).

### G6PD enzyme activity

G6PD enzyme activity was determined as described previously,^42^ and the activity assay used in this study has been described previously in detail.^21^

### NADPH level

The cultured cells were transfected with PAK4-short hairpin RNA and Flag-PAK4 plasmid. Then cells were incubated for an additional 24 h, collected and then measured using NADP+/NADPH Quantification Kit (Biovision, Mountain View, CA, USA).

### Palmitic acid, cholesterol and other metabolite level

Cells were seeded in culture plates until the cells reached 80–90% confluence. The cells were then collected and mailed to Shanghai minxin information technology company with dry ice. Different metabolites were measured by adopting GC–MS method.

### Patients

Human colon cancer tissues and paired-adjacent non-tumor colon tissues (5 cm away from the cancer edge) were obtained from 154 patients undergoing radical colon resection at the First Hospital of China Medical University. Fresh samples were snap frozen in liquid nitrogen immediately after resection and were stored at −80 °C. All samples were obtained with patients’ informed consent. The samples were histologically confirmed by staining with hematoxylin and eosin. The histological grade of cancer was assessed according to the criteria set by the World Health Organization. The tumor node metastasis classification was performed according to the standard criteria of the seventh tumor node metastasis staging system.

### Immunohistochemistry

Immunohistochemistry of cancer tissues as well as noncancerous tissues has been performed as described previously^43^ and immunohistochemical results were evaluated by HSCORE (histological score).^44^

### Statistics

All statistical analysis were performed using SPSS (17.0) software program (SPSS Inc, Chicago, IL, USA). *χ*^2^-test was used to determine the relationship between G6PD and PAK4 expression. Mann–Whitney U-test was used to determine significant differences between immunohistochemistry analysis results of colon cancer tissues and adjacent noncancerous tissues. For glucose consumption, G6PD enzyme activity and NADPH level, *t*-test was used to determine the significant differences between the treatment and control groups.

## Figures and Tables

**Table 1:** Different metabolites found in GC/MS chromatograms of the shRNA-control and shRNA-PAK4 groups

**Metabolite**	**Rt (min)**	**m/z**	**Chemical class**	**VIP**	***P*****-value**^a^
l-Threonine	9.95	181	Amino acid	1.30	0.02
Pyrimidine	11.07	240	Nucleotides	1.21	0.04
l—Lysine	12.57	518	Amino acid	1.35	0.01
Putrescine	12.68	174	Polyamine	1.34	0.02
Aspartic acid	12.83	232	Amino acid	1.38	0.02
l-Proline	12.86	230	Amino acid	1.51	0.00
l-Glutamic acid	12.86	66	Amino acid	1.47	0.01
d-Galactose	17.09	321	Carbohydrates	1.48	0.01
Palmitic acid	18.19	312	Fatty acid	1.45	0.01
Inositol	18.35	318	Vitamin	1.52	0.01
Cholesterol	27.90	368	Lipid	1.37	0.01
Cholesta-5,24-diene	28.29	456	Lipid	1.49	0.01

a Indicated statistical significance (*P*<0.05)

**Table 3:** G6PD expression during colon cancer progression

**Feature**	***n***	**G6PD expression**		***P*****-value**
		**Weak**	**Strong**	
*Age (years)*				
<65	84	32	52	0.390
⩾65	70	22	48	
				
*Gender*				
Male	82	33	49	0.152
Female	72	21	51	
				
*Tumor size (cm)*				
<5 cm	76	28	48	0.649
⩾5 cm	78	26	52	
				
*Histological grade*				
Poorly	9	3	6	0.042^a^
Moderately	116	35	81	
Well	29	16	13	
				
*Depth of invasion (pT)*				
T1,T2	22	11	11	0.114
T3,T4	132	43	89	
				
*Lymph node metastasis (pN)*				
No	105	39	66	0.430
Yes	49	15	34	
				
*Distant metastasis (pM)*				
No	139	50	89	0.475
Yes	15	4	11	
				
*Pathological stage (pStage)*				
Stages I, II	97	40	57	0.037^a^
Stages III, IV	57	14	43	

a Indicated statistical significance (*P*<0.05)

**Table 2:** PAK4 expression during colon cancer progression

**Feature**	***n***	**PAK4 expression**		***P*****-value**
		**Weak**	**Strong**	
*Age (years)*				
<65	84	20	64	0.388
⩾65	70	21	49	
				
*Gender*				
Male	82	23	59	0.670
Female	72	18	54	
				
*Tumor size (cm)*				
<5 cm	76	20	56	0.932
⩾ 5 cm	78	21	57	
				
*Histological grade*				
Poorly	9	2	7	0.003^a^
Moderately	116	24	92	
Well	29	15	14	
				
*Depth of invasion (pT)*				
T1,T2	22	5	17	0.656
T3,T4	132	36	96	
				
*Lymph node metastasis (pN)*				
No	105	30	75	0.425
Yes	49	11	38	
				
*Distant metastasis (pM)*				
No	139	38	101	0.543
Yes	15	3	12	
				
*Pathological stage (pStage)*				
Stages I, II	97	32	65	0.02^a^
Stages III, IV	57	9	48	

a Indicated statistical significance (*P*<0.05)

## Supplementary Material

Supplementary Figure s1-s3
