# Supplementary material for: PAK4 regulates G6PD activity by p53 degradation involving colon cancer cell growth
Source: Cell Death Dis. 2017 May 25;8(5):e2820–. doi: 10.1038/cddis.2017.85 (PMC5520749; doi:10.1038/cddis.2017.85)
Supplement: Supplementary Figure s1-s3 [file cddis201785x1.doc]

**Inventory.**

Fig. S1

Fig. S2

Fig. S3

**
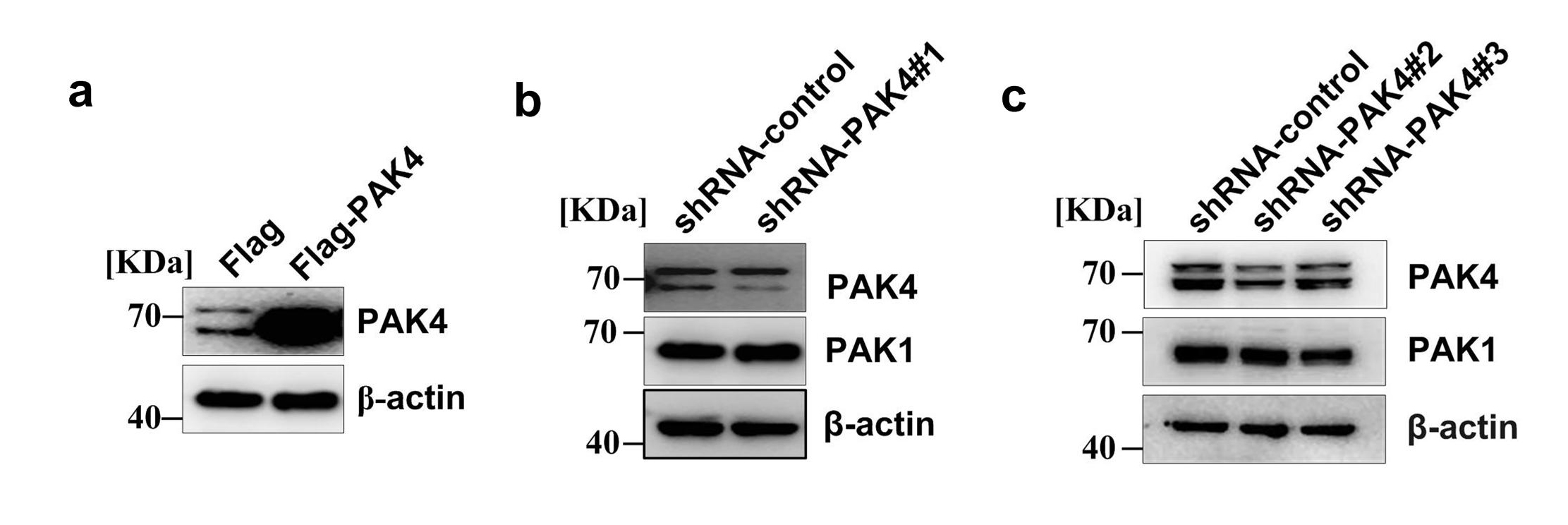
**

**Fig. S1 related to Fig. 2. PAK4 promotes consumption of glucose and NADPH production**

(a) HCT-116 p53+/+ cells were transfected with PAK4 plasmid.

(b) HCT-116 p53+/+ cells were stably transfected with lentiviral shRNA- PAK4#1 and the shRNA-control .

(c) HCT-116 p53+/+ cells were stably transfected with lentiviral shRNA- PAK4#2, shRNA- PAK4#3 and the shRNA-control

**
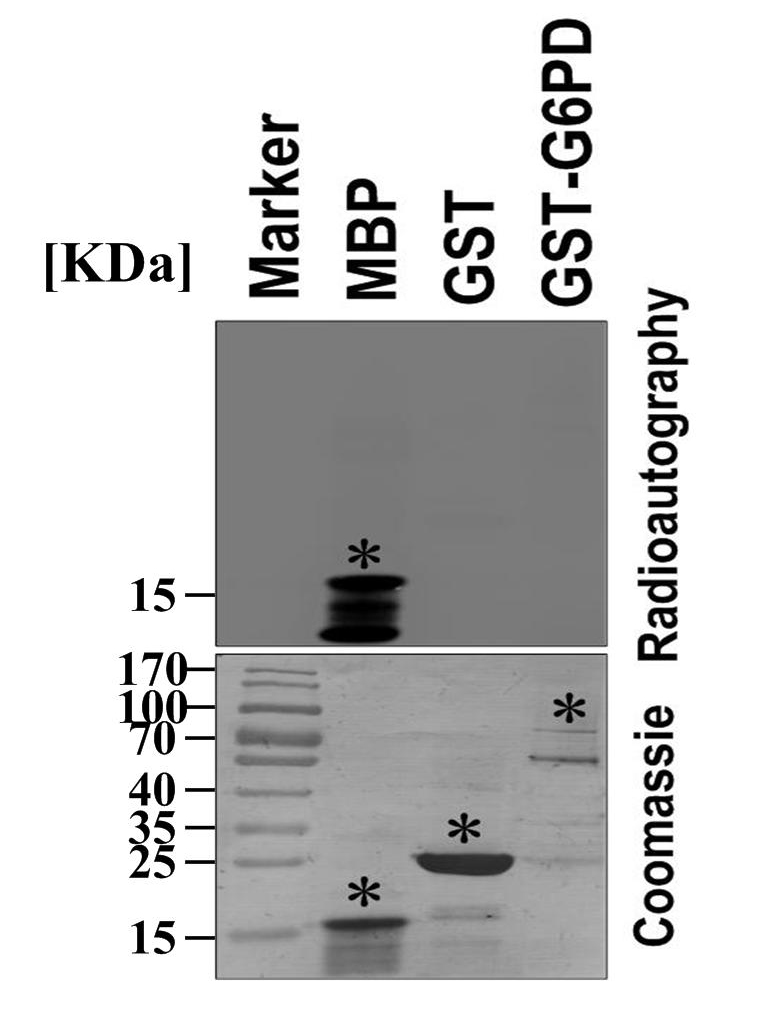
**

**Fig. S2, related to Fig. 4. Identification of G6PD as an interacting protein of PAK4**  PAK4 did not phosphorylate G6PD. In vitro kinase assay was performed using GST-tagged G6PD as a substrate, and catalytic active PAK4 as a kinase. Phosphorylated products were subjected to autoradiography and coomassie staining was presented under the image. Black stars indicate the MBP,GST or GST-G6PD.

**
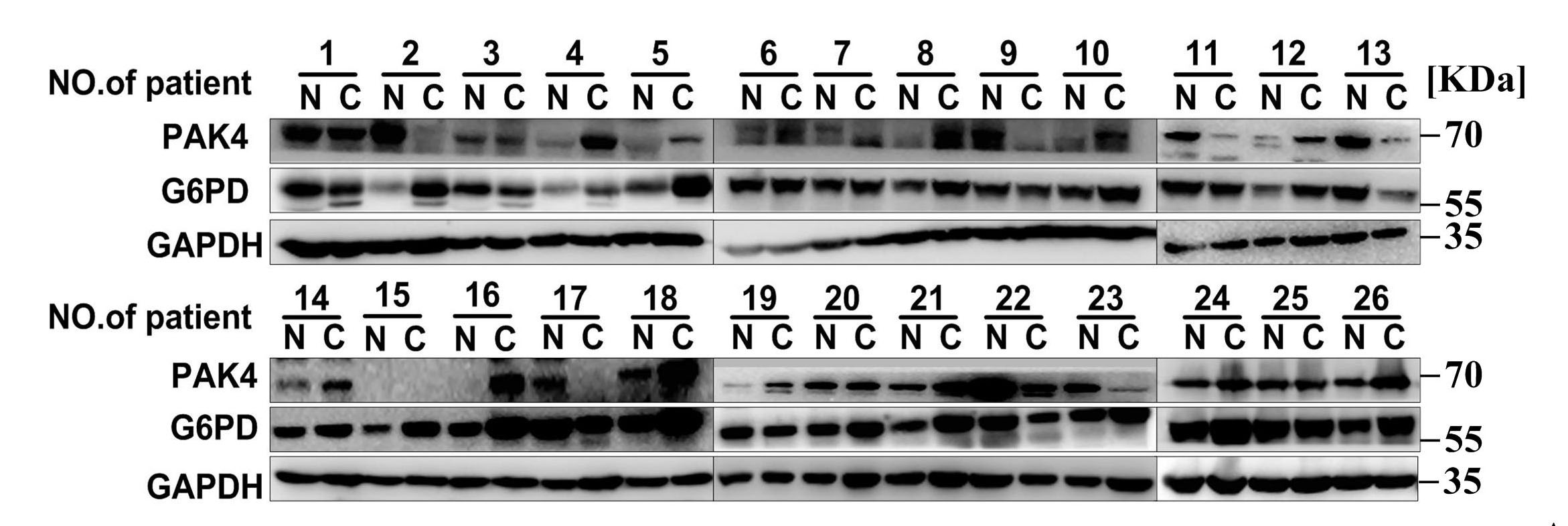
**

**Fig.S3, related to Fig. 7. Correlation of PAK4 and G6PD in colon cancer**

The protein levels of PAK4 and G6PD were examined in colon cancer. N, matched adjacent noncancerous mucosa. C, colon cancer tissue. GAPDH served as protein loading control,26 pairs are shown.
